# Decoupling of bacterial production and respiration in the surface water of the North Pacific Subtropical Gyre

**DOI:** 10.1007/s42995-025-00279-9

**Published:** 2025-04-02

**Authors:** Yuchen Zhang, Yibin Huang, Feipeng Xu, Shujie Cai, Yao Liu, Chao Xu, Lizhen Lin, Jixin Chen, Edward Allen Laws, Xin Liu, Bangqin Huang

**Affiliations:** 1 State Key Laboratory of Marine Environmental Science, Xiamen University, Xiamen, 361005 China; 2 College of the Environment and Ecology, Xiamen University, Xiamen, 361005 China; 3 College of the Ocean and Earth Sciences, Xiamen University, Xiamen, 361005 China; 4 Department of Environmental Sciences, College of the Coast and Environment, Louisiana State University, Baton Rouge, LA USA

**Keywords:** Bacterial metabolism, Bacterial production, Bacterial respiration, Community structure, Subtropical gyre

## Abstract

Heterotrophic bacterial production and respiration, two important contributors to carbon cycling, play an important role in global biogeochemical cycles. However, recent research suggests that these two processes may be decoupled, and the underlying changes in community structure and their interactions remain unclear. In this study, two research expeditions to the North Pacific Subtropical Gyre (NPSG) during the summer and winter of 2020–2021 revealed seasonal shifts in bacterial metabolism and community structure in response to environmental factors. The findings indicated notable seasonal fluctuations in bacterial abundance and production in the surface waters. Both peaked in winter compared to summer. Alterations in bacterial abundance that were further evident at the community level demonstrated significant seasonal differences in bacterial community structure and diversity and revealed, in particular, the intricacy of the networks and interactions among bacterial communities in winter. Bacterial respiration displayed no significant seasonal variations and was decoupled from bacterial abundance and production. The implication was that bacterial production did not directly dictate bacterial respiration. Specific taxa exerted a more substantial influence on bacterial respiration, potentially including groups with high respiration rates but relatively low abundance, thus challenging the notion that highly abundant taxa are invariably the most metabolically active. Moreover, the interplay between different bacterial taxa and their interactions may also impact the overall strength of bacterial community respiration. These findings significantly enhance our understanding of the decoupling between bacterial production and respiration, which is crucial for unraveling the complex mechanisms underlying carbon cycling and energy flow in marine ecosystems.

**Supplementary Information:**

The online version contains supplementary material available at 10.1007/s42995-025-00279-9.

## Introduction

Bacteria within microbial communities serve as critical players in carbon biogeochemical processes (Azam et al. 1983) and contribute significantly to the marine carbon cycle as essential components of the microbial food web. The metabolic pathways of bacteria are intricate systems shaped by the dynamic nature of environment. By mediating the flux of carbon and nutrients, these microorganisms influence climate regulation, oceanic productivity, and the health of marine ecosystems (Azam and Malfatti 2007; Ducklow et al. 2013). Knowledge of the metabolism and patterns of bacteria, and the connection to their community, is, therefore, of great significance for understanding the marine ecological processes that maintain ecosystem function and how the structure of microbial communities respond to environmental change at regional scales.

Bacterial metabolism encompasses two primary aspects: first, bacteria take up dissolved organic carbon (DOC) and convert it into biomass to form particulate organic carbon (POC), a process known as bacterial production (BP). This bacterial production plays a critical role in cycling nutrients within the microbial food web (Azam et al. 1983) and establishes connections between DOC and higher trophic levels (Kim et al. 2017). On average, bacterial production can account for 10–20% of primary productivity (Cole et al. 1988; Ducklow 2000). Conversely, bacteria derive energy by decomposing organic matter in the water column and releasing inorganic nutrients and CO_2_. Bacterial thus act as decomposers (Azam et al. 1983). Numerous studies have indicated that bacterial respiration (BR) can represent a substantial portion of community respiration (40 ~  > 90%) (Del Giorgio et al. 2002; Robinson 2008), making it a key process in carbon remineralization. The balance between anabolism and catabolism in bacterial metabolic processes has significant implications for global biogeochemical cycles (Ducklow et al. 2013). By regulating the flux of carbon and nutrients, these microbes help regulate climate, marine productivity, and the health of marine ecosystems (Hutchins and Fu 2017).

Traditional perspectives suggest that in extensive marine systems, bacterial production, respiration, and abundance (BA) exhibit coherent variations over broad temporal and spatial scales (Del Giorgio et al. 1997; Bouvier et al. 2007). However, this paradigm has been challenged by recent findings that have found a seeming decoupling between BA and BR, with significant differences in respiration rates among bacterial, where most respiration is attributed to rare lineages, while dominant bacterial species exhibit minimal respiration rates (Munson et al. 2022). Diverse bacterial taxonomic groups may possess varied metabolic capabilities and efficiencies that influence organic carbon processing within the community and within respiratory pathways (Korlević et al. 2016). Consequently, changes in community structure, particularly those involving minor taxa, could prove pivotal for BR, as alterations in the relative abundances of specific bacterial groups may lead to a decoupling between BA and respiration rates (Munson et al. 2022).

Microbial communities interact in complex ways and aggregate into ecological modules based on different phylogenetic relationships and environmental preferences (Lurgi et al. 2019; Xia et al. 2015). Microorganisms rely on complex interactions between internal members to maintain stability and resilience to environmental disturbances (Wu et al. 2021). Recent studies based on network approaches have revealed the significant impact of environmental interference on the patterns of microbial co-occurrence (complexity and modularity) (Zhang et al. 2021). These co-occurrence network analyses have helped to not only elucidate how the complexity of microbial communities respond to changes in environmental factors but also to reveal how connections between microorganisms affect ecosystem functions (Feng et al. 2021; Qiu et al. 2021).

Subtropical gyres are typical oligotrophic environments with very low surface chlorophyll *a* (Chl *a*) concentrations and have traditionally been regarded as an ocean desert (Dai et al. 2023). In such low-nutrient systems, the efficiency of energy transfer to higher trophic levels is characteristically low (Kletou and Hall-Spencer 2012). The importance of the microbial loop is even greater in these oligotrophic systems because of the utilization of dissolved organic matter (DOM) by heterotrophic bacteria (Azam and Malfatti 2007). However, it is still unclear how seasonal changes in oligotrophic marine ecosystems affect the complexity of bacterial interactions and how changes within key modules of bacterial communities influence bacterial metabolism. The aim of this study was to: (1) elucidate the spatiotemporal variations of bacterial metabolism in the North Pacific Subtropical Gyre (NPSG), a typical oligotrophic region of the Pacific Ocean, and (2) in conjunction with bacterial community structure and network relationships, seek to explore the potential reasons for the decoupling between BP and BR, as well as its implications for understanding marine microbial ecology and carbon cycling.

## Materials and methods

### Study sites, sampling, and environmental parameters

**Fig. 1:**
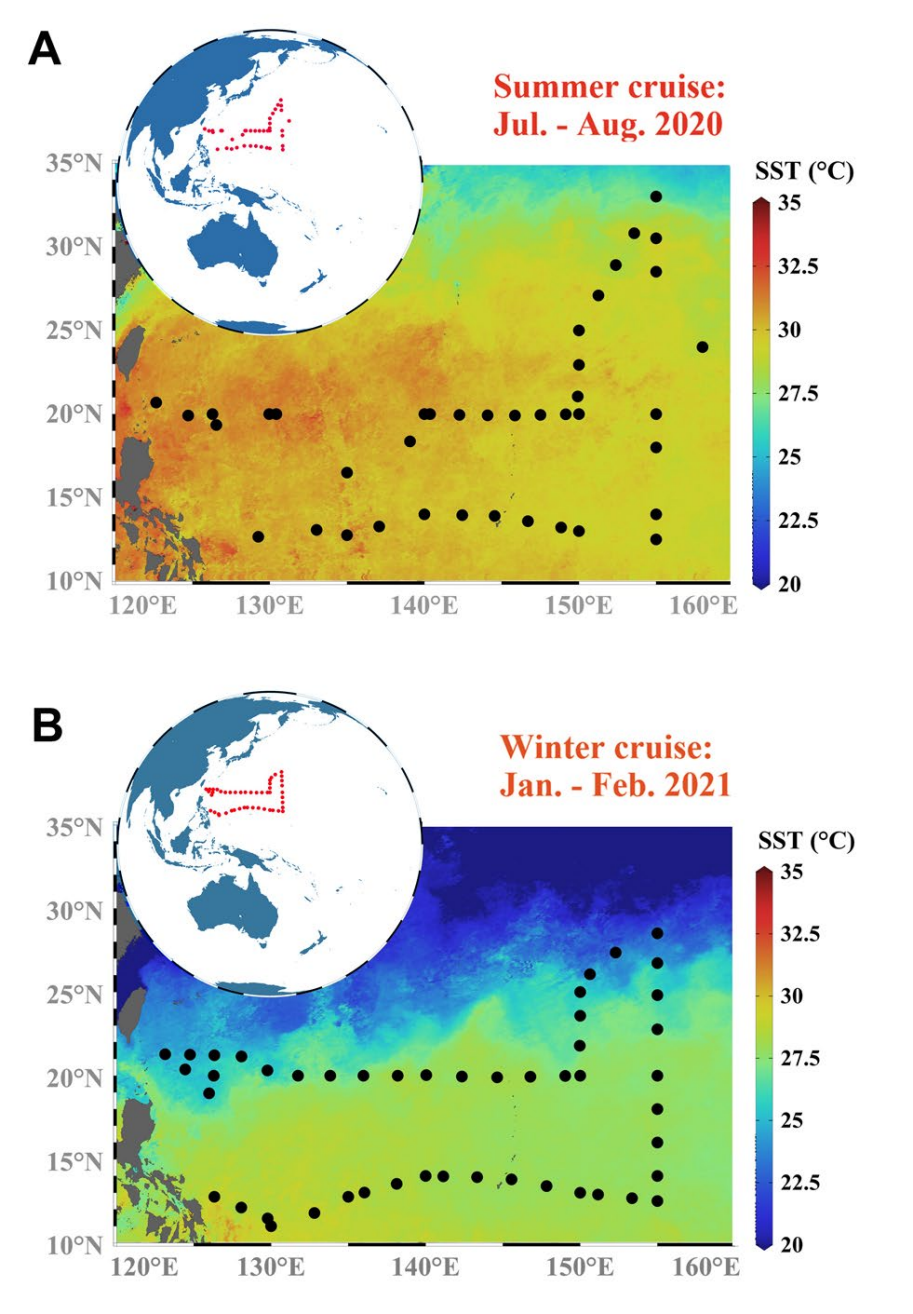
Map of sampling stations in the North Pacific Subtropical Gyre. **A** 40 stations were sampled during the KK2003 summer cruise in 2020. **B** 48 stations were sampled during the KK2007 winter cruise in 2021. The sea surface temperature (SST, °C) of the base map is based on 2 month average of the AQUA_MODIS sampling period (July, August 2020 and January, February 2021, respectively)

This study was conducted in the NPSG on the R/V *Tan Kah Kee* during the GEOTRACES GPpr15 (KK2003 and KK2007) cruises: the KK2003 summer cruise from 3 July to 24 August 2020 and the KK2007 winter cruise from 22 December 2020 to 7 February 2021. A total of 40 and 48 stations were investigated, respectively, on these two cruises (Fig. 1). Twelve-liter Niskin sampling bottles alongside a conductivity–temperature–depth (CTD) were used to collect surface seawater samples. The seawater temperature and salinity at each station were measured using a Sea-Bird Electronics CTD SBE 911plus probe. Samples for the measurement of Chl *a* concentration were collected by filtering 500 mL of seawater through 25-mm GF/F filters (Whatman), which were then stored in liquid nitrogen. In the laboratory, the samples were submerged in 90% acetone in a dark environment at  - 20 °C for 16–24 h until analysis with a Trilogy fluorometer (Turner Designs, USA) (Welschmeyer 1994). The NO_3_, PO_4_ and SiO_4_ data at each station were obtained from World Ocean Atlas 2018 using 3-D extrapolation based on the geographic location, sampling month and depth. These data provided the monthly averages of nutrient concentrations (Boyer et al. 2018).

### Bacterial abundance, production, and respiration

For bacterial-abundance sample, 1.8 mL of seawater was collected after a 20-µm prefiltration. The samples were first transferred to a 2-mL centrifuge tube and fixed by adding 20 µL of 50% paraformaldehyde before temporary storage in liquid nitrogen and then transfer to a  - 80 °C freezer. In the laboratory, 300 µL of the sample after thawing was used for staining with SYBR Green and placed in a flow cytometer (BD Accuri C6) for measurement (Marie et al. 1997).

Bacterial production was determined using a modified protocol of the ^3^H-leucine incorporation method (Kirchman 1993). Four 1.8 mL aliquots of water were collected from each sample and added to 2 mL sterile microcentrifuge tubes. They were incubated with a saturating concentration (10 nmol L^−1^) of ^3^H-leucine (Perkin Elmer, USA) for 2 h in the dark at the in-situ temperature. One sample was immediately killed by adding 100% trichloroacetic acid (TCA) as a control, and the other three incubations were stopped by the addition of TCA at the end of the 2-h incubation. Samples were filtered onto 0.2-μm polycarbonate filters and then rinsed twice with 5% TCA and three times with 80% ethanol before being stored at  – 80 °C. In the laboratory, the filters were transferred to scintillation vials with 5 mL of Ultima Gold scintillation cocktail. The incorporated ^3^H-leucine was determined using a Tri-Carb 2800TR liquid scintillation counter (Perkin Elmer, USA). The leucine–carbon conversion factor uses 0.37 kg C mol^−1^ leucine to convert bacterial productivity from the rate of leucine uptake to the rate of carbon assimilation, bacteria production = Leu Incorporation × 0.37 kg C mol^−1^ Leu. (Huang et al. 2019).

Bacterial respiration was estimated based on in vitro INT reduction rates as described by Martínez‐García et al. (2009). Four 200 mL polypropylene plastic bottles were filled with seawater. One bottle was immediately fixed by adding formaldehyde (2% final concentration) as a blank. Fifteen minutes later, the four replicates were inoculated in the dark by the addition of 2-para (iodophenyl)−3(nitrophenyl)−5(phenyl) tetrazolium chloride tetrazolium salt (INT) at a final concentration of 0.8 mM. The INT samples were incubated at the in-situ temperature. After incubations of 2 h, the reactions were stopped by adding formaldehyde. All the samples were sequentially filtered after 15 min through 0.8-μm and 0.2-μm pore size polycarbonate filters and stored frozen until further processing. The INT-f reduction rates were measured using a SP 8001 UV/Vis Spectrophotometer at 485 nm. The concentration of INT-f on the membrane was calculated via a standard curve made from the INT-f standard. To transform INT reduction rates into O_2_ consumption, we used an empirical relationship of logCR_O2_ = 0.72logINT_T_ + 0.44 (García-Martín et al. 2019). Bacteria respiration = CR_O2_ × INT_0.2–0.8_/INT_T_. O_2_-based BR was then converted into carbon units assuming that the respiratory quotient (O:C) was 1 (Hopkinson 1985). Cell-specific bacterial production (csBP) and cell-specific bacterial respiration (csBR) are the single cell rate obtained by dividing BP and BR, respectively, by BA.

### Bacterial community

DNA samples were obtained by filtering 20 L of surface seawater via a 0.22-μm Millipore filter (PC, Millipore, Billerica, MA, USA) using a peristaltic pump. The seawater was first filtered through a 200-μm bolting cloth to remove non-target organisms. Each filter was placed in a cryovial and frozen at  - 80 °C until extraction. DNA was extracted using the DNeasy PowerWater Kit (Qiagen, Germany). Samples were stored at  - 80 °C until amplification with PCR.

The bacterial 16S rRNA V3 and V4 fragments were amplified by the polymerase chain reaction at 94 °C for 2 min followed by 25–27 cycles of amplification (94 °C for 30 s, 55 °C for 30 s, and 72 °C for 60 s), and a final step of 72 °C for 10 min. We used the primers 341F (5'-CCTACGGGNGGCWGCAG-3') and 805R (5'-GACTACHVGGGTATCTAATCC-3') (Herlemann et al. 2011) and then carried out sequencing using the Illumina NovaSeq 6000 sequencer. The raw read sequences were processed in QIIME2 (Bolyen et al. 2019). The adaptor and primer sequences were trimmed using the cutadapt plugin. The DADA2 plugin was used for quality control and to identify amplicon sequence variants (ASVs) (Callahan et al. 2016). It generated a total of 58,093 ASVs. Taxonomic assignments of representative ASV sequences were performed with a confidence threshold of 0.8 by a pre-trained Naive Bayes classifier that was trained on the Greengenes version 13.8. Representative sequences were then blasted against the Silva132 database (Yilmaz et al. 2014) for taxonomic assignment. For further analysis, ASVs assigned to chloroplasts and mitochondria were removed. The ASV table in our study was rarefied to 49,920 based on the values of the minimum sequence numbers to normalize the sampling effort. R software was used for community composition analysis. All the sequences obtained from this study have been submitted to the National Center for Biotechnology Information (NCBI) Sequence Read Archive (SRA) database under the accession number PRJNA1105212.

### Co-occurrence network construction

All the samples were used to construct the co-occurrence networks using the WGCNA package in R software (version 4.3.1) (Langfelder and Horvath 2012). To reduce interference from low abundance samples, the rarefied ASVs that occurred in less than 5 samples were excluded (Gao et al. 2021). The pairwise Spearman correlation matrices among ASVs were calculated, and type I error rates (*p* values) were corrected using the Benjamini Hochberg’s correction (Benjamini et al 2006). Nodes with correlations greater than 0.75 and *p* < 0.001 were retained (Delgado‐Baquerizo et al. 2018). The network topological parameters (including modularity, average clustering coefficient, average path length, and average degree) and degree centrality were extracted using the “igraph” R package. The software Gephi (version 0.10.1) was performed to visualize the final cooccurrence networks and the relative abundance of each module in all networks was calculated from the average standardized relative abundance of the species (z-score).

### Statistical analyses

For each sample, bacterial alpha diversity indices, including the Shannon and Richness indices, were calculated in the R package “vegan”, and the microbial beta diversities were compared based on the Bray–Curtis dissimilarity matrices between samples. The nonparametric Wilcoxon test was performed to evaluate the differences in environmental characteristics (temperature, salinity, and Chl *a*), bacterial metabolism (BA, BP and BR), bacterial alpha diversity indices (Richness and Shannon), and beta diversity among cruises in different seasons. Differences with *p* < 0.05 were considered to be significant. We conducted normality and homogeneity of variance tests on the values of our environmental variables and found that some did not meet these assumptions. We, therefore, chose non-parametric methods because of their robustness to assess differences without relying on distributional assumptions. An unconstrained principal coordinate analysis (PCoA) was used to reveal the differences in bacterial community composition among seasons, and the permutation multivariate analysis of variance (PERMANOVA) test was carried out to explain the statistical significance of the differences. We then performed principal component analysis (PCA) using the “vegan” package in R to visualize the distribution pattern of the shifts in BR and the properties of the bacterial communities. We also conducted a linear regression analysis to evaluate the relationships between BR and the relative abundance of the main modules in the networks.

## Results

### Environmental characteristics

**Fig. 2:**
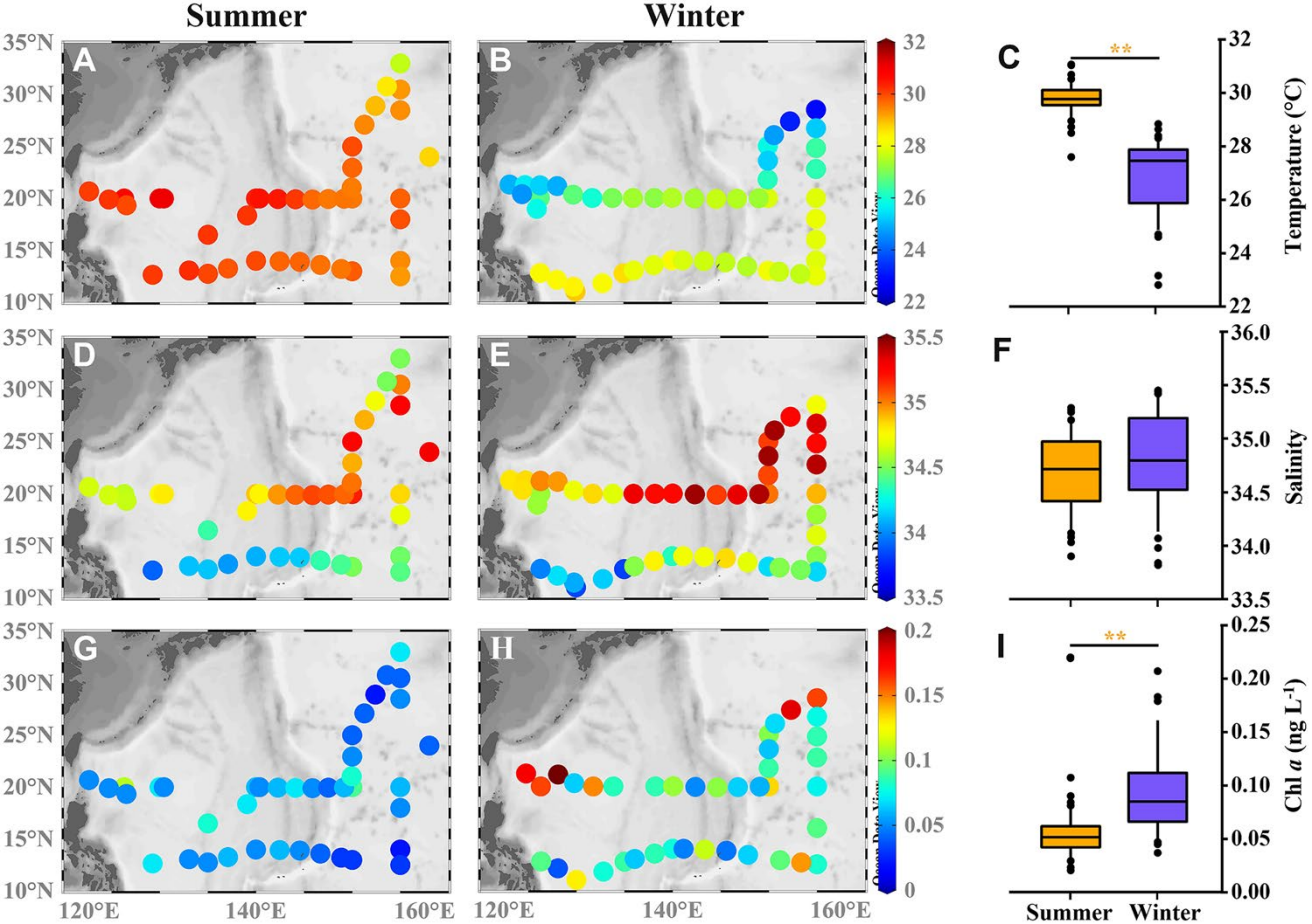
Horizontal distribution of surface temperature (**A**-**C**), salinity (**D**-**F**) and chlorophyll *a* (Chl *a*) (**G-I**) in the surface water of NPSG (Wilcoxon test, **p < 0.001). NPSG denoted North Pacific Subtropical Gyre

The average sea surface temperature in summer was 29.8 ± 0.6 °C, with a range of 27.6–31.1 °C (Fig. 2A–C). It was slightly lower to the east of 12°N and 20°N sections than in the west, and it gradually decreased from south to north along 155°E sections. The average surface temperature in winter was 26.9 ± 1.4 °C, and the range was 22.8–28.8 °C. The temperature change was larger in winter than in summer. It gradually increased from west to east along the 20°N section, but it gradually decreased from west to east along the 12°N section, and it gradually decreased from south to north as a whole. Compared with the two seasons, the sea surface temperature was significantly higher in summer than in winter (Fig. 2C), and the temperature difference from south to north gradually increased. The range of the temperature was larger in winter than in summer, and the distribution pattern was similar in the two seasons.

The average salinity of surface water in summer was 34.7 ± 0.4, and the range was 33.9–35.3 (Fig. 2D–F). Salinity gradually increased from west to east along the 12°N and 20°N sections, and it gradually increased from south to north. The salinity was higher along the 20°N section than the 12°N section. The average salinity of surface water in winter was 34.8 ± 0.5, and the range was 33.8–35.5, which was no different from that in summer. The characteristics of the distribution of salinity were also consistent in winter and summer: salinity gradually increased from west to east and from south to north.

The mean Chl *a* concentration in surface water in summer was 0.05 ± 0.02 μg L^−1^, and the range was 0.02–0.11 μg L^−1^ (Fig. 2G–I). The distribution of Chl *a* concentrations in surface water in summer was relatively uniform, and the range was small. In winter, the north–south distribution was different: the average concentration was 0.10 ± 0.04 μg L^−1^, and the range was 0.04–0.21 μg L^−1^, which was significantly higher than that in summer. Therefore, although the concentrations of Chl *a* in this region were low, there was still a significant seasonal pattern. The nutrient concentrations (NO_3_, PO_4_, SiO_4_) in surface water were generally low in both summer and winter. Only PO_4_ showed a significant seasonal variation (Fig. S1).

### Bacterial metabolic activities

**Fig. 3:**
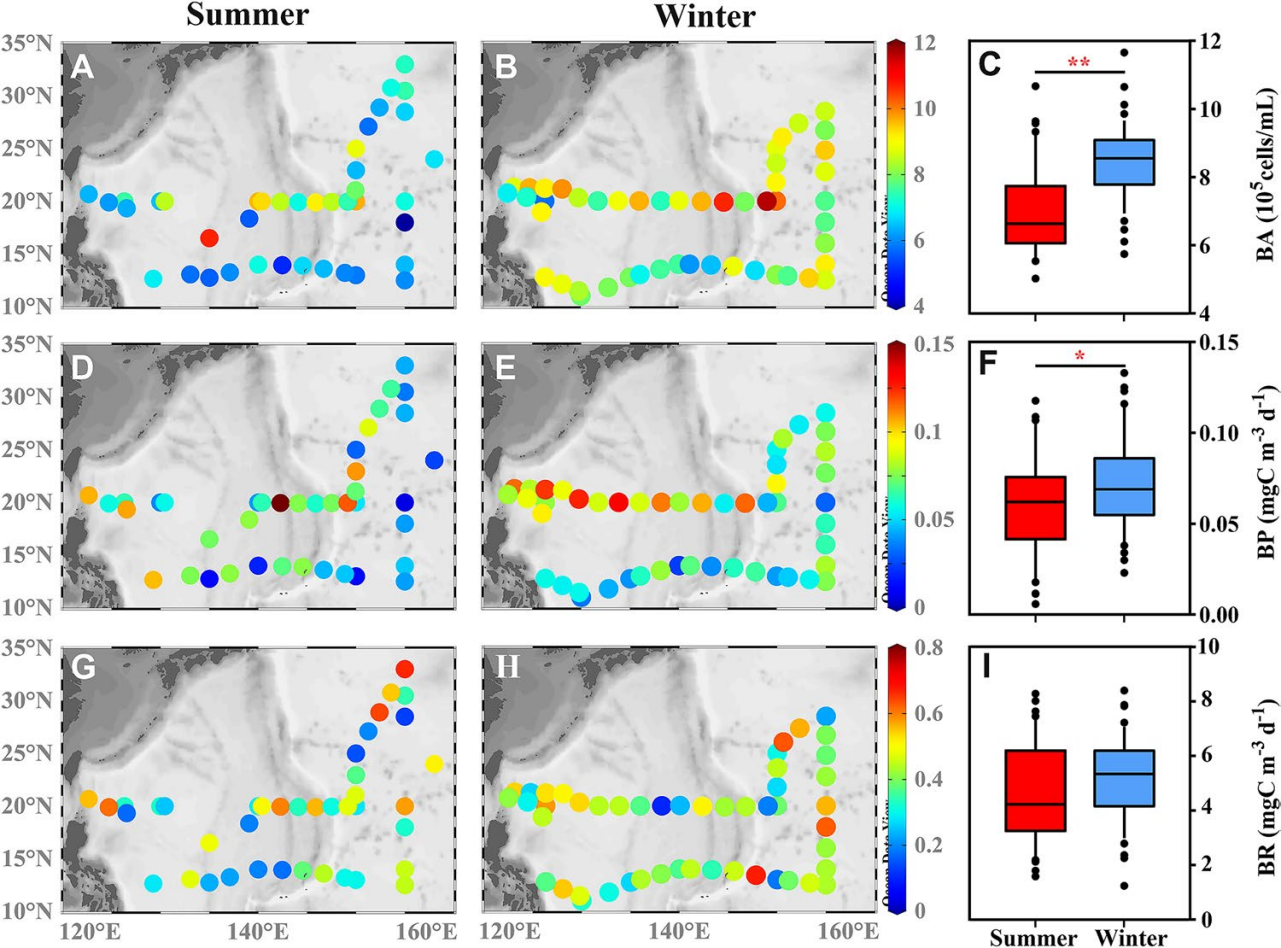
Horizontal distribution of bacterial metabolism in surface water including bacterial abundance (BA) (**A**-**C**), bacterial production (BP) (**D**–**F**) and bacterial respiration (BR) (**G**–**I**) (Wilcoxon test, *p < 0.05, **p < 0.001)

BA at the surface exhibited significant seasonal variations (Fig. 3A–C). The BA in summer was 6.95 ± 1.49 × 10^5^ cells mL^−1^, with a range of 1.49–10.67 × 10^5^ cells mL^−1^. In winter, the BA was 8.45 ± 1.13 × 10^5^ cells mL^−1^, and the range was 1.13–11.65 × 10^5^ cells mL^−1^, which was significantly higher than that in summer (Fig. 3C).

The BP at the surface in summer was 0.06 ± 0.04 mg C m^−3^ d^−1^, with a range of 0.01–0.19 mg C m^−3^ d^−1^. In contrast, during winter, it increased slightly to 0.07 ± 0.03 mg C m^−3^ d^−1^, with a range of 0.02–0.13 mg C m^−3^ d^−1^. Similar to BA, BP also underwent significant seasonal fluctuations, with higher values in winter compared to summer (Fig. 3D–F).

Unlike BA and BP, BR at the surface did not undergo significant seasonal changes; there were no significant differences between winter and summer (Fig. 3G–I). The BR in summer was 4.56 ± 1.80 mg C m^−3^ d^−1^, with a range of 1.56–7.92 mg C m^−3^ d^−1^. In winter, the BR was 4.92 ± 1.44 mg C m^−3^ d^−1^, with a range of 1.2–8.04 mg C m^−3^ d^−1^. Most of the spatial differences in bacterial metabolism within the sampling area were not statistically significant between the two seasons (see Supplementary Figs. S3 and S4).

### Bacterial community composition and diversity

**Fig. 4:**
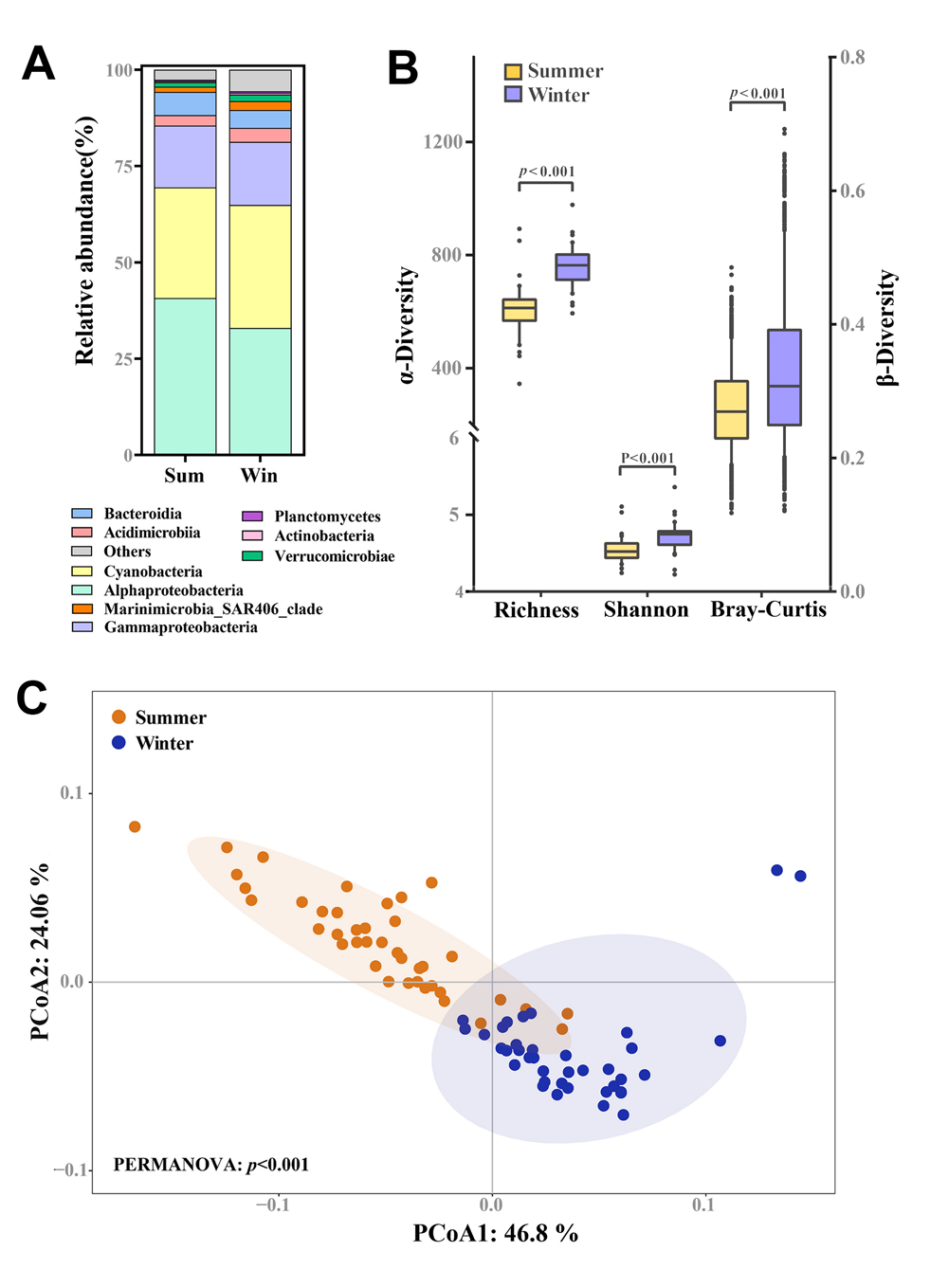
Bacterial community in the surface water. **A** Relative abundance (%) of the dominant bacterial. **B** Boxplot comparing mean values of alpha (Richness and Shannon) and beta diversity indices (Bray–Curtis). **C** Unconstrained principal coordinates analysis (PCoA) shows that the bacterial communities of each season have significantly distinct microbiota as detected by permutational multivariate analysis of variance (PERMANOVA)

Obvious differences in the bacterial community composition were apparent among two seasons (Fig. 4). At the class level, the dominant group in summer were Alphaproteobacteria, Cyanobacteria, and Gammaproteobacteria. They accounted for 40.7%, 28.7%, and 16.0% of the total community composition, respectively. In winter, the proportion of Alphaproteobacteria decreased to 32.9%, that of Cyanobacteria increased to 31.9%, and that of Gammaproteobacteria (16.4%) did not change significantly (Fig. 4A). The α-diversity indices of bacterial communities including the Richness and Shannon indices were significantly higher in winter (Wilcoxon test, *p* < 0.001). Specifically, compared with summer, the Richness and Shannon indices of the bacterial community in winter increased by 24.6% and 5.0%, respectively. Results based on the Bray–Curtis dissimilarity index revealed that the beta-diversity of the bacterial community was significantly higher in winter than in summer (Wilcoxon test, *p* < 0.001) (Fig. 4B). The PCoA results showed that the bacterial community compositions during the two seasons were significantly different, and the PERMANOVA further verified that this difference was statistically significant (*p* < 0.001) (Fig. 4C).

### Bacterial co-occurrence networks

**Fig. 5:**
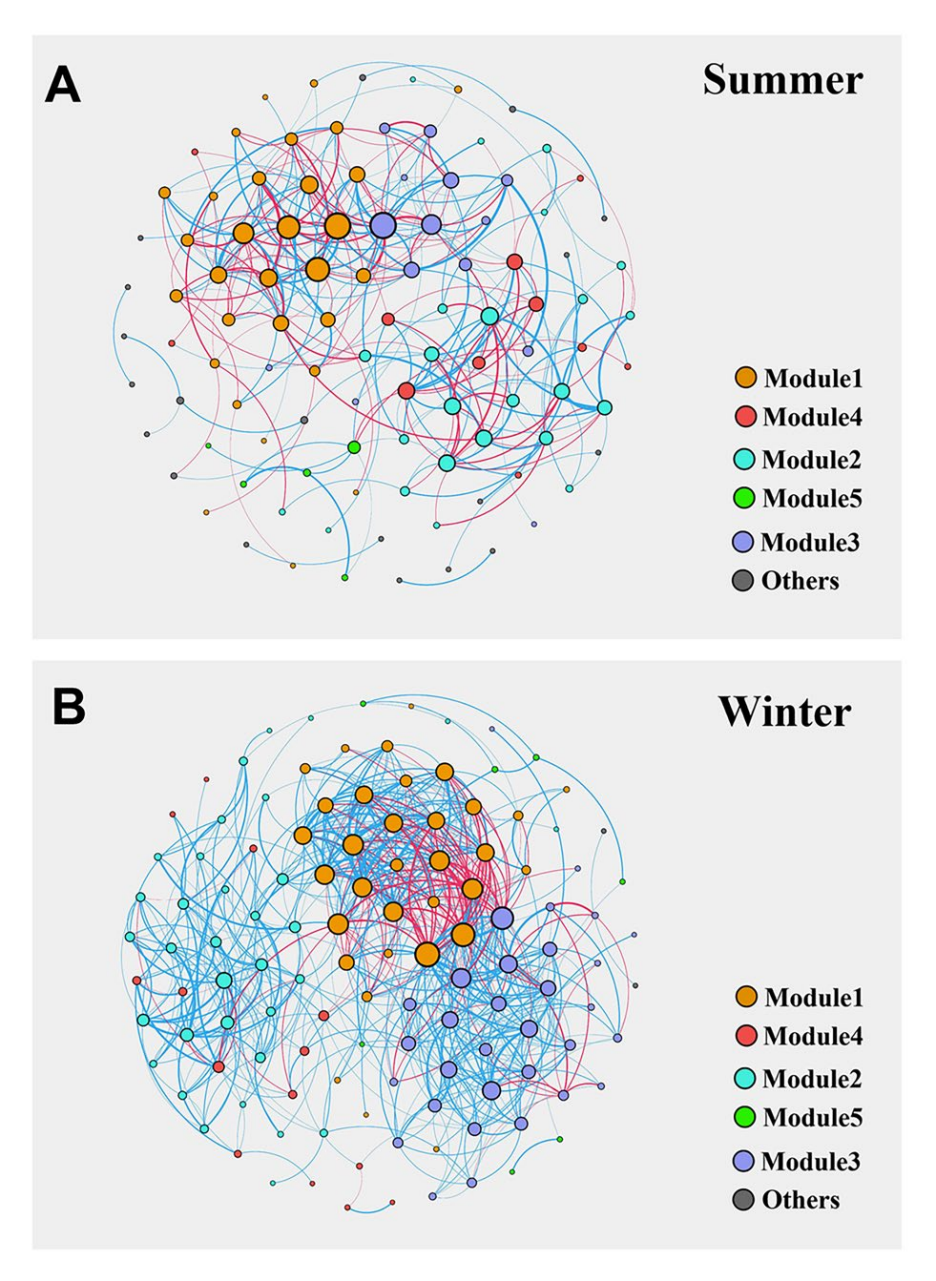
Co-occurrence networks of bacterial communities in the surface water. A connection represents a strong (Spearman correlation threshold *R* >|0.75|) and significant (*p* < 0.01) correlation. For each panel, the size of each node is proportional to the quantity of connections (degree), and the thickness of each connection between two nodes (edge) is proportional to the value of Spearman’s correlation coefficients

A co-occurrence network was constructed for each season based on the strong correlations among ASVs (Fig. 5). These two networks in Fig. 5 are colored based on their modular class. The red edges show negative interactions between two bacterial nodes, while the blue edges show positive interactions between two nodes. Each node represents a bacterial species. The results of the basic structure of each network showed that there were differences in the co-occurrence networks of bacterial communities in the two seasons (Table 1). The number of shared edges was much higher in summer (657) than in winter (307) (Table 1). The summer network had higher modularity and average path length. The mean degree was the same in winter and summer. The degree centrality and average clustering coefficient were higher in winter than in summer. These results indicated that the structure of the bacterial community network was more complex and the interaction between communities was stronger in winter.

**Table 1:** Major topological properties of the co-occurrence networks of bacterial communities in the surface water

	Nodes	Edges	Modularity	Average clustering coefficient	Average Path length	Average degree	Degree centralization
Summer	103	301	0.501	0.429	2.272	5.845	0.149
Winter	121	657	0.476	0.542	1.969	10.860	0.201

### Relationships between bacterial respiration and bacterial community structure

**Fig. 6:**
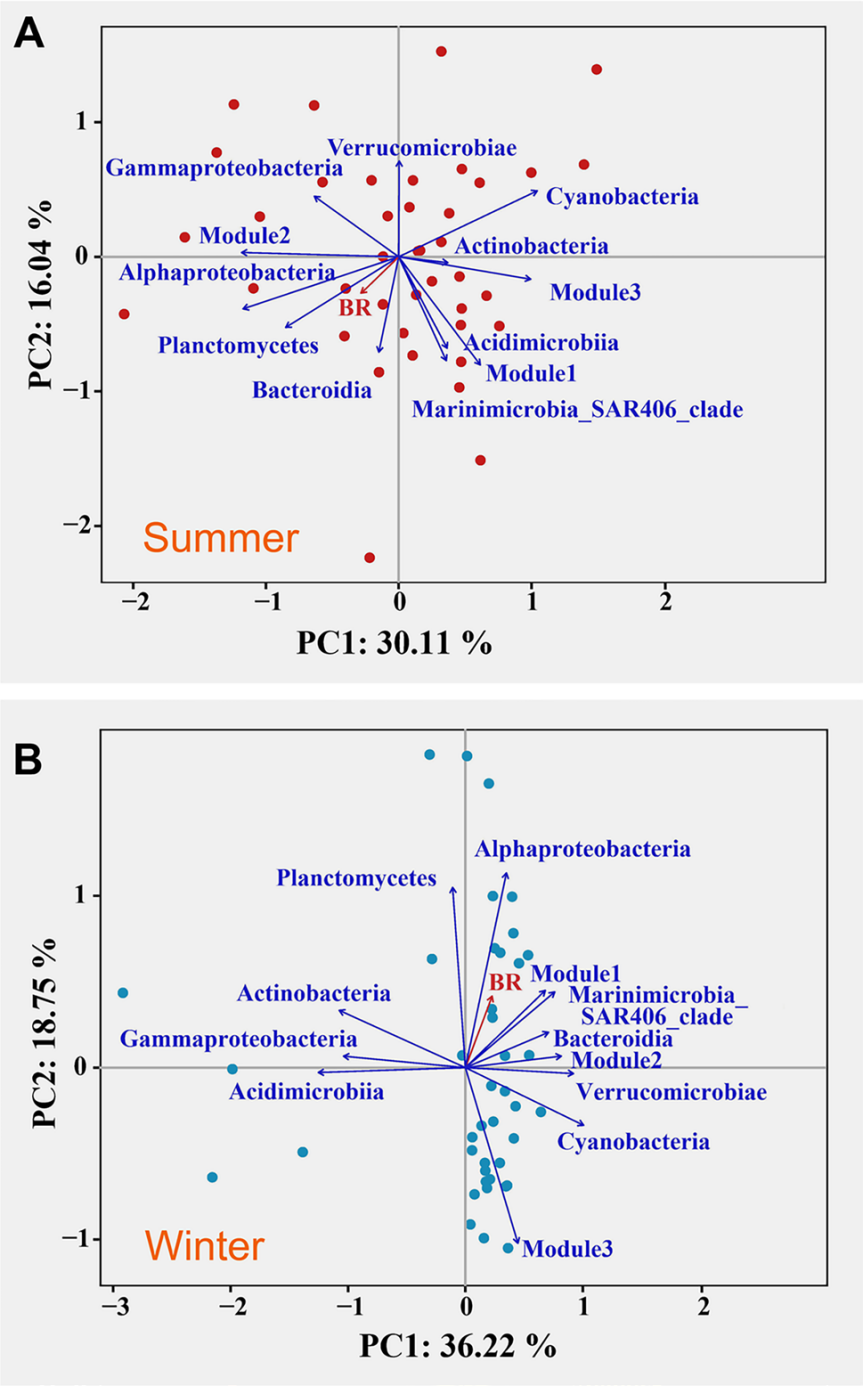
Principal component analysis (PCA) shows the linkage between bacterial respiration and shifts in the properties of the bacterial community (the relative abundance of dominant class-level taxa, as well as the relative abundance of the three main ecological clusters in the co-occurrence network) among the surface water of summer and winter

A PCA was conducted for each season to elucidate the relationships between BR and the properties of the bacterial community structure (Fig. 6). The first two principal axes explained 46.15% and 54.97% of the cumulative percentage variance for summer and winter, respectively. For the dominant taxa, the relative abundances of Alphaproteobacteria were positively correlated with BR in both seasons (Fig. 6). In summer, the relative abundances of Bacteroidia and Planctomycetes were also potential drivers of the variations of BR (Fig. 6A). In winter, the relative abundances of Bacteroidia, Planctomycetes, and the Marinimicrobia_SAR406_clade were positively associated with BR (Fig. 6B).

**Fig. 7:**
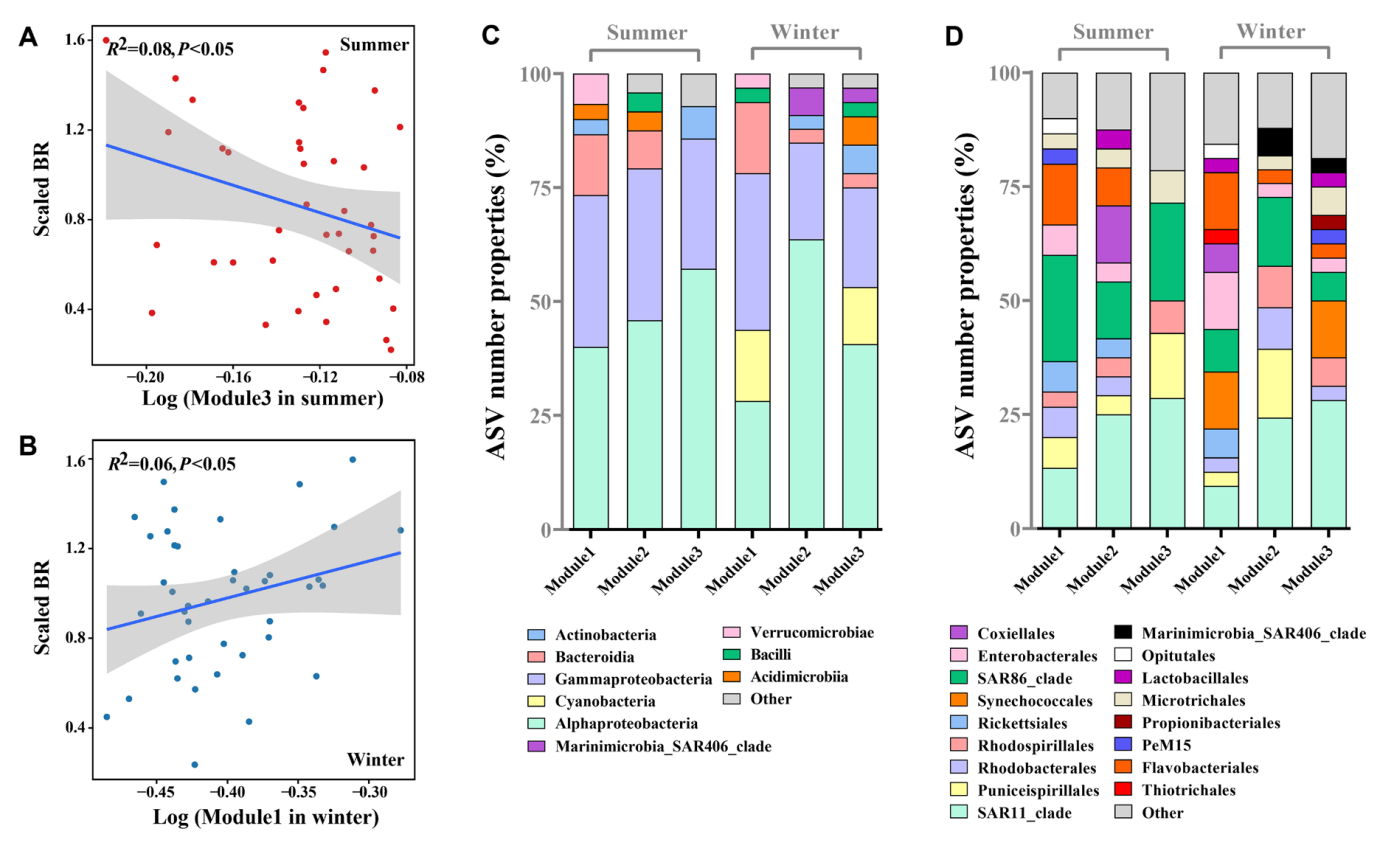
Linear regressions between the scaled BR (value divided by the maximum of all values) and the relative abundance of Module 3 in summer (**A**) and Module1 in winter (**B**) (only significant cases are shown in the figures). Amplicon sequence variants (ASVs) number properties of the dominant class (**C**) and order (**D**) level taxa in the three main ecological clusters among summer and winter

We explored the relationship between BR and the relative abundance of the top three key microbial assemblages with the highest number of nodes in each network. Significant negative associations between the relative abundance of module 3 and BR in summer were found via both the PCA (Fig. 6A) and the linear regression analysis (*R*^2^ = 0.08, *p* < 0.05) (Fig. 7A). The key assemblages for community composition of Module 3 in summer were dominated by Alphaproteobacteria (57.14%), Gammaproteobacteria (28.57%), and Actinobacteria (7.14%) at the class level (Fig. 7C) and by SAR11_clade (28.57%), SAR86_clade (21.43%), Puniceispirillales (14.29%), Rhodospirillales (7.14%) and Microtrichales (7.14%) at the order level (Fig. 7D). Strong and significant positive correlation between the relative abundance of module 1 and BR in winter were determined via both the PCA (Fig. 6B) and linear regression analysis (*R*^2^ = 0.06, *p* < 0.05) (Fig. 7B). The dominant bacterial combinations of module 1 in winter at the class level were Gammaproteobacteria (34.38%), Alphaproteobacteria (28.13%) and Bacteroidia (15.63%) (Fig. 7C). At the order level, Flavobacteriales (12.50%), SAR11_clade (9.38%), SAR86_clade (9.38%), Rickettsiales (6.25%), Rhodobacterales (3.13%) and Thiotrichales (3.13%) were found to be dominant (Fig. 7D).

## Discussion

### Effects of seasonal evolution of environmental parameters on bacterial metabolism in subtropical regions

Our findings corroborated a markedly higher Chl *a* concentration during winter compared to summer (Fig. 2I). Research indicates that Chl *a* concentrations at Station ALOHA also exhibit notable seasonal fluctuations. That site experiences relatively high Chl *a* concentrations during winter compared to summer (Karl et al. 2001). Several factors contribute to this phenomenon, including oceanic physical processes, nutrient dynamics, and ecological interactions (Letelier et al. 1996). For instance, the winter cooling and subsequent convective mixing transport nutrient-rich deep waters to the surface, augmenting nutrient availability that stimulates phytoplankton growth and subsequent increases of Chl *a* (Polovina et al. 2008; Wu et al. 2017; Yamada et al. 2004). Moreover, diminished metabolic rates and alterations in community structure may mitigate grazing pressure from zooplankton and facilitate proliferation of the phytoplankton population (Rivkin et al. 2001).

Previous studies have revealed that the BA in surface waters at Station ALOHA typically ranges from approximately 10^5^ to 10^6^ cells mL^−1^, and it undergoes seasonal fluctuations that may lead to temporary increases of bacterial populations, especially in response to nutrient inputs or changes in environmental conditions (Karl et al. 2014). In our study, BA fell within the reported ranges for the NPSG region (Liu et al. 2002), and it underwent seasonal variations, with notably higher levels in winter compared to summer even in this oligotrophic environment (Fig. 3C). The elevated winter Chl *a* concentrations compared to summer, along with their significant positive correlation with BA, highlight the importance of phytoplankton primary production in driving the increase of BA. In oligotrophic regions of the Northwestern Pacific, cyanobacteria like *Prochlorococcus* and *Synechococcus* dominate the phytoplankton community in surface waters (Imai et al. 2002). The DOC produced by cyanobacteria under nutrient-limited conditions consists primarily of labile low-molecular-weight DOC that is rich in amino acids (Berman and Holm-Hansen 1974; Karl et al. 2001). The labile nature of this DOC supports heterotrophic bacterial growth, enhances microbial metabolism, and contributes to carbon cycling within the upper ocean layers (Azam et al. 1983; Carlson 2002).

**Fig. 8:**
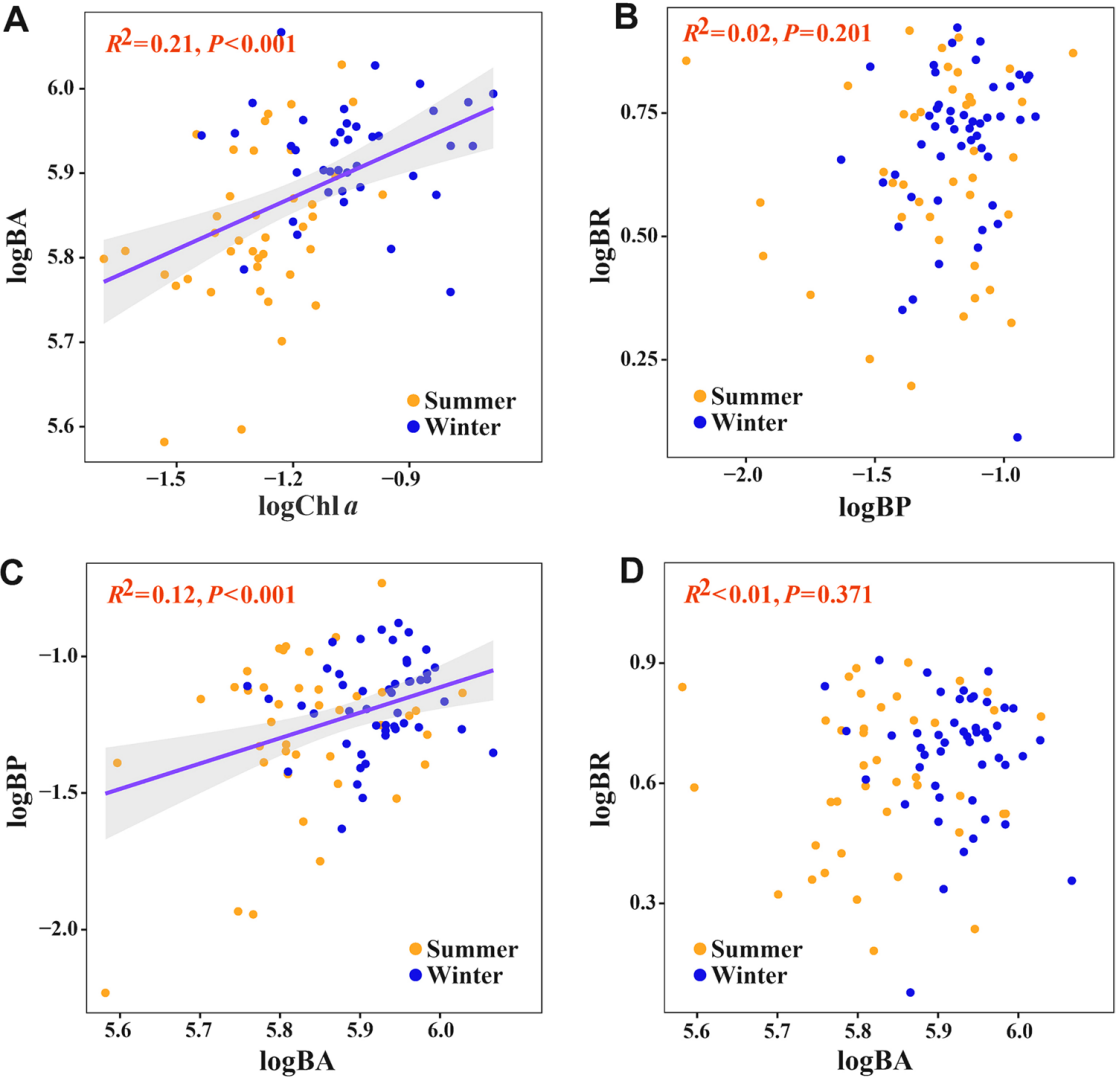
Relationship between various variables. Chl *a*, BA, BP and BR denoted chlorophyll *a*, bacterial abundance, bacterial production, and bacterial respiration, respectively

**Fig. 9:**
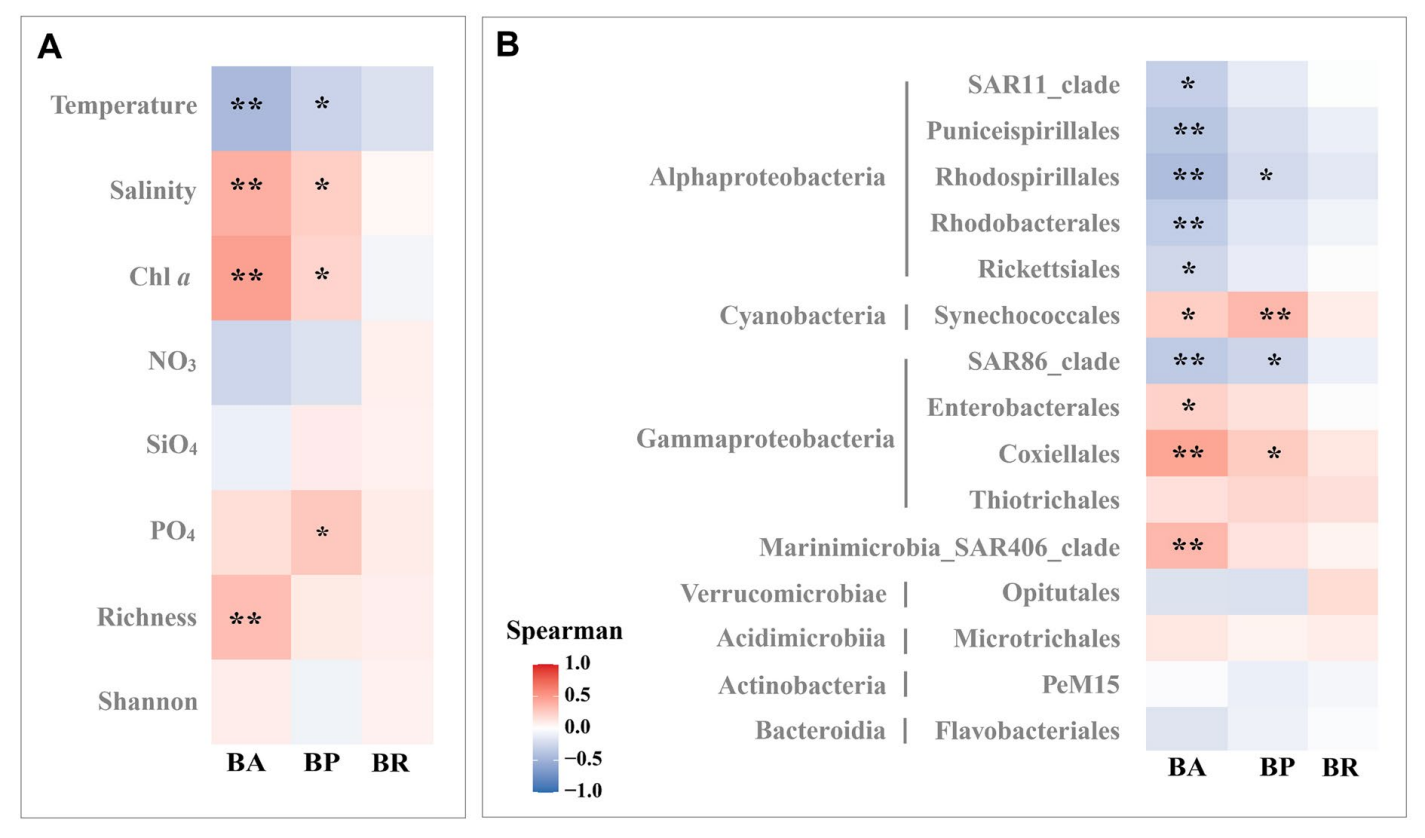
Heatmap of statistical relationships between (**A)** bacterial metabolism and environmental factors and alpha diversity, (**B)** bacterial metabolism and bacterial community in the order level (*p < 0.05, **p < 0.01)

In addition, a significant correlation between BP and BA (Fig. 8C) was observed, with no significant difference of csBP between winter and summer (Fig. S2A). The indication was that the increase of BP stemmed primarily from the elevated BA. The increase of BP in winter and its significant correlation with Chl *a* concentrations (Figs. 2I and 9A) suggest that the enhanced BP could also be attributed to the increase of phytoplankton biomass. A coupling of Chl *a* concentrations and BP has been documented in other regions as well (Gomes et al. 2015; Xu et al. 2018). The significant positive correlation between Synechococcales and BA as well as BP (Fig. 9B) similarly indicates that increased Chl *a* in winter implies more phytoplankton growth that leads to the production of more labile DOC. This, in turn, stimulates bacterial utilization, promotes bacterial proliferation, and enhances BP. Seasonal fluctuations and environmental factors play a significant role in shaping the relationship between BA and BR in the North Pacific Gyre (Church et al. 2008). Previous studies have indicated that temperature profoundly and directly influences heterotrophic processes and metabolic activities in planktonic communities (Fenchel 2005). However, the Southern Ocean study did not document a correlation between temperature and respiration rates (Schapira et al. 2009). Interestingly, our results also did not reveal a significant correlation between temperature and BR (Fig. 9A). The implication was that BR in the NPSG may simply be insensitive to temperature in the range of temperatures (23–31 °C) in this study. The increase of phytoplankton biomass was expected to provide a supply of labile DOC from phytoplankton. This supply is considered to be a crucial factor in regulating the respiration rate of heterotrophic bacteria (Xu et al. 2018).

### Linkage between bacterial production and respiration

Bacterial production and consumption of carbon are important processes in marine ecosystems, because they are among the key determinants of the metabolic state of the oligotrophic ocean. Over the past few decades, the metabolic state of oligotrophic regions has been a topic of ongoing debate. Net community production obtained through incubation methods has suggested that most oligotrophic regions are heterotrophic. This scenario is inconsistent with the positive exports of organic matter determined via incubation-free methods or sediment traps (Duarte et al. 2013; Ducklow et al. 2013; Williams et al. 2013). BRs are typically estimated by measuring changes in dissolved oxygen concentrations in situ or during laboratory cultivation experiments, often over periods of 24 h or longer (Pomeroy et al. 1994). Previous studies have indicated that changes of bacterial community composition (Massana et al. 2001; Gattuso et al. 2002), nutrient depletion (Ram et al. 2003), and measurement inaccuracy (Guo et al. 2022) may have compromised the results of these extended cultivation experiments. In this, we used the in vitro reduction of INT, a metric of electron transport system activity, to estimate BR (García-Martín et al. 2019).

Several assumptions underlie the INT method. First, the INT reduction method relates to the toxicity of INT to living cells (Martínez-García et al. 2009). However, this toxicity occurs only after a certain length of time after the addition of INT and production of formazan. The validity of the method, therefore, requires that maximum incubation times be shorter than the time when toxicity becomes manifest. We conducted experiments to determine the maximum incubation time during two research cruises, and our results indicated that the 2-h incubation time we used was within the range of maximum incubation times for the sampling area (Fig. S5). A second problem inherent to the limitations of the method is the possible reduction of INT not associated with cellular respiration (Martínez-García et al. 2009). When calculating the rates of INT reduction, we subtracted the absorbance of the control from the absorbance of the live, incubated samples to ensure that the final rate of INT reduction was not biased by this absorbance (García-Martín et al. 2019). Finally, how does the INT method compare with the Winkler method? The CR_O2_-INT_T_ conversion equation derived in García-Martín et al. (2019) was successfully validated with an independent data set. We conducted parallel sampling using both methods at selected stations during both cruises. The results indicated a strong correlation between these two methods (Fig. S6). Despite the reliance of both methods on certain assumptions, the INT method is generally considered to be more accurate and efficient. The uncertainties associated with the INT method did not significantly influence our conclusions. In the future, alternative methods such as high-precision dissolved oxygen probes on autonomous platforms (e.g., Biogeochemical Argo floats and Seagliders) may be utilized to better assess respiration rates (Xu et al. 2024).

The traditional view of a strong correlation between BP and BR in the marine environment has been shaped by concepts of carbon turnover, the assumption of steady state, and simplified models (Carlson et al. 2009; Ducklow et al. 2000; Giovannoni et al. 2005). They have typically depicted a linear relationship between BP and BR on the assumption that changes of one process would directly affect the other. However, in our study, the absence of a significant correlation between BP and BR (Fig. 8B), suggested that the two processes varied incoherently, i.e., there was a decoupling between BP and BR. In addition, studies have also suggested that the decoupling of BP and BR in surface waters of the oligotrophic NPSG was influenced by nutrient limitation, microbial community dynamics, seasonal variability, and ecosystem interactions (Church et al. 2005; Karl et al. 2008; Karl and Church 2014). In this study, the observed increase of the concentration of BA during winter may have been the primary driver of the elevated BP (Figs. 3C and 8C). Moreover, BA and BP were decoupled from BR (Fig. 8B, D). Further analysis of the correlations between BR and microbial modules in winter and summer, alongside the identification of distinct bacterial taxa and combinations within modules (Fig. 7), underscored the fact that BA alone did not determine BR. We hypothesize that the difference between BA, BP, and BR is one of the mechanisms responsible for the decoupling of BP and BR. In future work, we will focus on the biogeochemical regulatory mechanisms, such as the type of DOM available.

In oligotrophic marine ecosystems, BP and BR directly impact carbon fluxes within these ecosystems (Carlson et al. 2007). The coupling of these two processes reflects a balance between organic matter production and subsequent decomposition. That coupling ensures the efficient utilization of available organic matter, promotes rapid turnover of carbon within the microbial loop, enhances energy transfer through the marine food web, and boosts the efficiency of the biological pump (Boyd et al. 2015). In oligotrophic areas, bacterial metabolic processes play a crucial role in transforming and remineralizing organic matter that is ultimately exported as a vertical flux of carbon particles (Hansell et al. 2009). As global climate change impacts ocean temperature, stratification, and nutrient cycling, the coupling between BP and BR may undergo changes (Robinson et al. 2010). When BP becomes decoupled from BR, a complex dynamic ensues that affects the efficiency of carbon transfer within the microbial loop and subsequently impacts the entire marine food web (Carlson et al. 2007). Decoupling between BP and BR may lead to reduced carbon export to deeper ocean waters and affect the efficiency of the biological pump in sequestering carbon in the deep ocean (Ducklow et al. 2000).

### Potential impact of bacterial community in decoupling bacterial production and respiration

The relationship between BA and BR is influenced by the community composition and functional diversity of microbial assemblages in the oligotrophic NPSG (Karl and Church 2014). Empirical evidence has suggested a similar reality, where increased abundance can foster greater microbial diversity (Ducklow 2000; Sarmento and Gasol 2012). In a comparable 6-year time series study of the Western English Channel, Gilbert et al. (2012) have reported that bacterial diversity peaks in winter and troughs in summer. Moreover, modeling of global marine bacterial richness also predicts diversity peaks in winter at temperate–subtropical latitudes (Ladau et al. 2013). In our study, the diversity in winter significantly surpassed that in summer (Fig. 4B), The notable positive correlation between BA and diversity indicated a substantial improvement in the diversity of bacterial communities in winter (Fig. 9A). Sarmento and Gasol (2012) have observed that a substantial proliferation of phytoplankton in oligotrophic waters leads to increased BA and diversity. In oligotrophic surface waters, increased BA might enhance ecosystem resilience by broadening the pool of functional traits capable of responding to shifts in environmental conditions (Rocca et al. 2015).

In contrast, Polymenakou et al. (2005) have demonstrated that in oligotrophic regions, bacteria exhibit extensive enzymatic activity that suggests that they utilize a wide range of organic substrates. This metabolic diversity implies that an increase of bacterial abundance may be accompanied by an increase in niche and substrate diversity. In addition, predation pressure also plays an important role in regulating the relationship between bacterial abundance and diversity. Predators including bacteriophages and protists exert top-down control on bacterial populations that may prevent any single species from achieving numerical dominance (Thingstad and Lignell 1997). This selective predation can maintain or even increase microbial diversity and allow competitively weak species to survive in the shadow of predators. The causal relationship behind bacterial abundance and bacterial community diversity is unclear. What we mainly observe are correlations and some indications, which may help to suggest cause and effect. In the future, a thorough analysis using metabolomics will be necessary to identify key taxa. That evidence will be critically important for understanding the impact of environmental changes.

Furthermore, alongside diversity, increased BA profoundly shapes the structure and intricacy of bacterial co-occurrence networks (Fig. 5 and Table 1). Co-occurrence networks delineate the relationships and interactions among various microbial taxa. Research utilizing network analysis has consistently demonstrated that as microbial communities become more diverse, the complexity of their interaction networks tends to amplify (Faust and Raes 2012; Fuhrman 2009). Each bacterial taxon may engage in distinct interactions, including competition or mutualism, and thereby contribute to the overall network complexity (Lurgi et al. 2019). For example, Barberán et al. (2012) have shown that microbial communities exhibit dense networks of both positive and negative associations. These associations imply a tightly interconnected ecosystem, where changes to one taxon could cause reverberations throughout the community. Increased BA can foster greater microbial diversity and thereby enrich the complexity of bacterial co-occurrence networks. This result reflects the intricate interactions supporting ecosystem stability and function (Louca et al. 2018).

In our investigation, BR, unlike BA and BP, exhibited no significant seasonal differences between summer and winter (Fig. 3), and it appeared to be decoupled from BA and BP (Fig. 8B, D). However, conspicuous seasonal variations were observed in bacterial community structure and diversity (Fig. 4), and there was a notable elevation in the complexity of bacterial co-occurrence networks during winter (Table 1). The relationship between BA, BR, and bacterial community structure may thus be highly intricate. Previous studies have indicated that respiration rates in the surface waters of the Northwest Pacific Ocean during autumn mirror those observed in spring (Odate 1995). Investigations in the Southern Ocean have demonstrated that respiration rates show no correlation with total BA (Schapira et al. 2009). Moreover, single-cell respiration and genomic analyses conducted on samples from geographically diverse locations in the coastal Gulf of Maine, the Atlantic, and the Pacific suggest a prevalent decoupling of respiration rates from cell abundance among marine prokaryoplankton taxa (Munson et al. 2022). Our results likewise indicated that there is a significant, positive correlation between the abundance of some bacterial taxa, such as Enterobacterales and Coxiellales, both members of the Gammaproteobacteria, and BA, but no correlation with BR (Fig. 9B). The succession of bacterial groups is related to their substrate preference (Xu et al. 2018). Alphaproteobacteria generally prefer low-molecular-weight DOC, such as amino acids (Cottrell and Kirchman 2000), whereas Bacteroidetes are composed of bacteria that generally utilize high-molecular-weight (HMW) DOC, such as polysaccharides, chitin, cellulose, and protein (Teira et al. 2011).

Bacterial communities are inherently complex assemblages comprising numerous species with varying metabolic rates (Servais et al. 2001). An insightful approach for studying microbial interactions involves detecting temporal co-occurrence patterns among phylotypes and visualizing them as association networks (Steele et al. 2011). In our study, we compared and analyzed the correlation between each module of the bacterial co-occurrence network and BR during two seasons. During summer, BR exhibited a negative correlation with the relative abundance of module 3 (Fig. 7A), primarily composed of SAR11 within the Alphaproteobacteria and SAR86 within the Gammaproteobacteria (Fig. 7D). These results suggested that higher proportions of symbiosis or associative combination patterns in the network were associated with lower BR. Notably, the SAR11 genus (*Pelagibacter*) and two genera of Gammaproteobacteria cluster SAR86 (AAA076-P13 and D2472) collectively constituted 22–24% of all cells but accounted for only 0.6–5.6% of the total respiration (Munson et al. 2022). In contrast, during winter, BR increased with the rise in the relative abundance of module 1, as indicated by the results of PCA analysis, which showed a positive correlation between the two groups (Figs. 6B and 9B). The proportion of SAR11 and SAR86 within module 1 during winter was significantly lower, with a more complex combination of bacterial taxa, including Rhodobacterales and Thiotrichales, both of which are Gammaproteobacteria (Fig. 7D). The implication is that higher proportions of patterns associated with larger groups in the network are linked to higher BR. Recent research has highlighted that some genera such as ASP10-02a and *Thioglobus* (Gammaproteobacteria), LFERO1 and *Planktomarina* (both members of the *Roseobacter* clade, Rhodobacteraceae, Alphaproteobacteria), and multiple genera of Bacteroidia contribute 10–37% of the total O_2_ consumption while comprising only 1.2–2.5% of cells (Munson et al. 2022).

In addition, the functional redundancy of respiration rates in marine bacteria plays a pivotal role in shaping microbial community dynamics and interactions (Louca et al. 2018). By harboring a diverse array of metabolic capabilities, marine bacteria can occupy distinct ecological niches and coexist within complex microbial networks (Hutchins et al. 2017). Whereas BA is indeed a crucial factor in marine microbial ecology, it does not by itself dictate the rates of BR. These findings suggest that BR may be significantly influenced by specific groups, some of which may exhibit high respiration rates despite low relative abundance. The implication is that high-abundance groups do not necessarily correspond to high metabolic activity. Moreover, the differing correlations between bacterial community composition patterns and rates of BR in the winter and summer modules suggest that different combinations of bacterial taxa and their interactions may also influence the overall respiration rates of the bacterial community.

## Conclusion

In this study, we conducted a comprehensive investigation of bacterial metabolism, community composition, diversity, and co-occurrence networks in the NPSG during the summer of 2020 and the winter of 2021. Our findings revealed significant seasonal variations of BA and BP within the surface waters of the NPSG. Both metrics were notably higher during winter compared to summer. Surprisingly, BR rates displayed no significant seasonal differences. The observed increase in the concentration of phytoplankton Chl* a* during winter was suggested to be the primary driver behind the elevated BA. Concurrently, the rise of BP stemmed from an increase of BA and shift of Chl* a*.

Moreover, the fact that changes of BA were mirrored at the community level elucidated significant seasonal differences in bacterial community structure and diversity. These alterations contributed to heightened complexity in co-occurrence networks and interactions among bacterial communities during winter. Notably, BA and BP were found to be decoupled from BR, with no discernible correlation between temperature and respiration rate. The implication was that temperature may not be the primary driver of spatiotemporal heterogeneity of respiration rates across diverse environments. Further analysis of correlations between BR and different modules in winter and summer, along with the identification of distinct bacterial taxa and combinations within modules, underscored the fact that BA alone does not dictate BR. Rather, specific taxa, potentially those with high respiration rates but relatively low abundance, exert greater influence. This highlights the fact that highly abundant taxa may not always be the most metabolically active. In addition, the disparate correlations observed between bacterial community composition patterns in winter and summer modules and BR suggest that interactions among different combinations of bacterial taxa may also shape the overall strength of BR.

Overall, our findings deepen understanding of seasonal variations in surface bacterial metabolism in the oligotrophic regions of the Northwest Pacific Ocean. Furthermore, they contribute to an enhanced understanding of the decoupling between BP and BR and thereby shed light on the intricate dynamics within marine microbial ecosystems.

## Supplementary Information

Below is the link to the electronic supplementary material.

Supplementary file1 (DOCX 910 KB)

## Data Availability

Data will be made available on request.
