# Supplementary material for: Decoupling of bacterial production and respiration in the surface water of the North Pacific Subtropical Gyre
Source: Mar Life Sci Technol. 2025 Apr 2;7(2):397–412. doi: 10.1007/s42995-025-00279-9 (PMC12102442; doi:10.1007/s42995-025-00279-9)
Supplement: Supplementary file 1 — Supplementary file1 (DOCX 910 KB) [file 42995_2025_279_MOESM1_ESM.docx]

Decoupling of bacterial production and respiration in the surface water of the North Pacific Subtropical Gyre

**Supplementary materials**


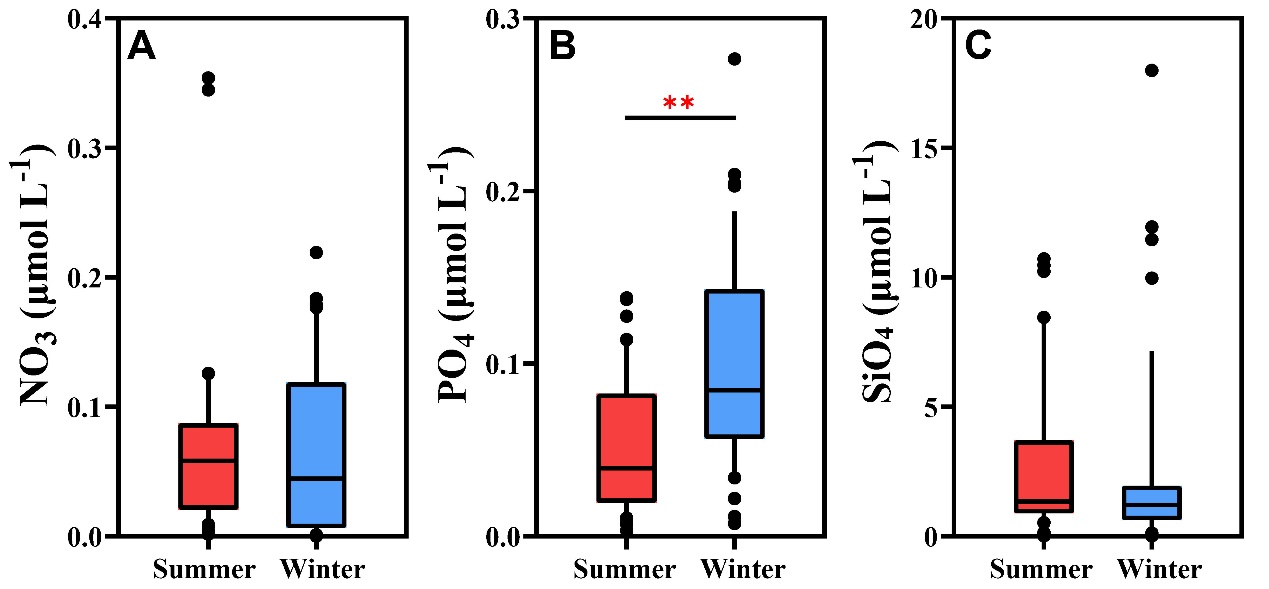


Fig. S1 The concentrations of nutrients between summer and winter include NO_3_ (A), PO_4_ (B), and SiO_4_(C) (Wilcoxon test, ** *p* < 0.001)


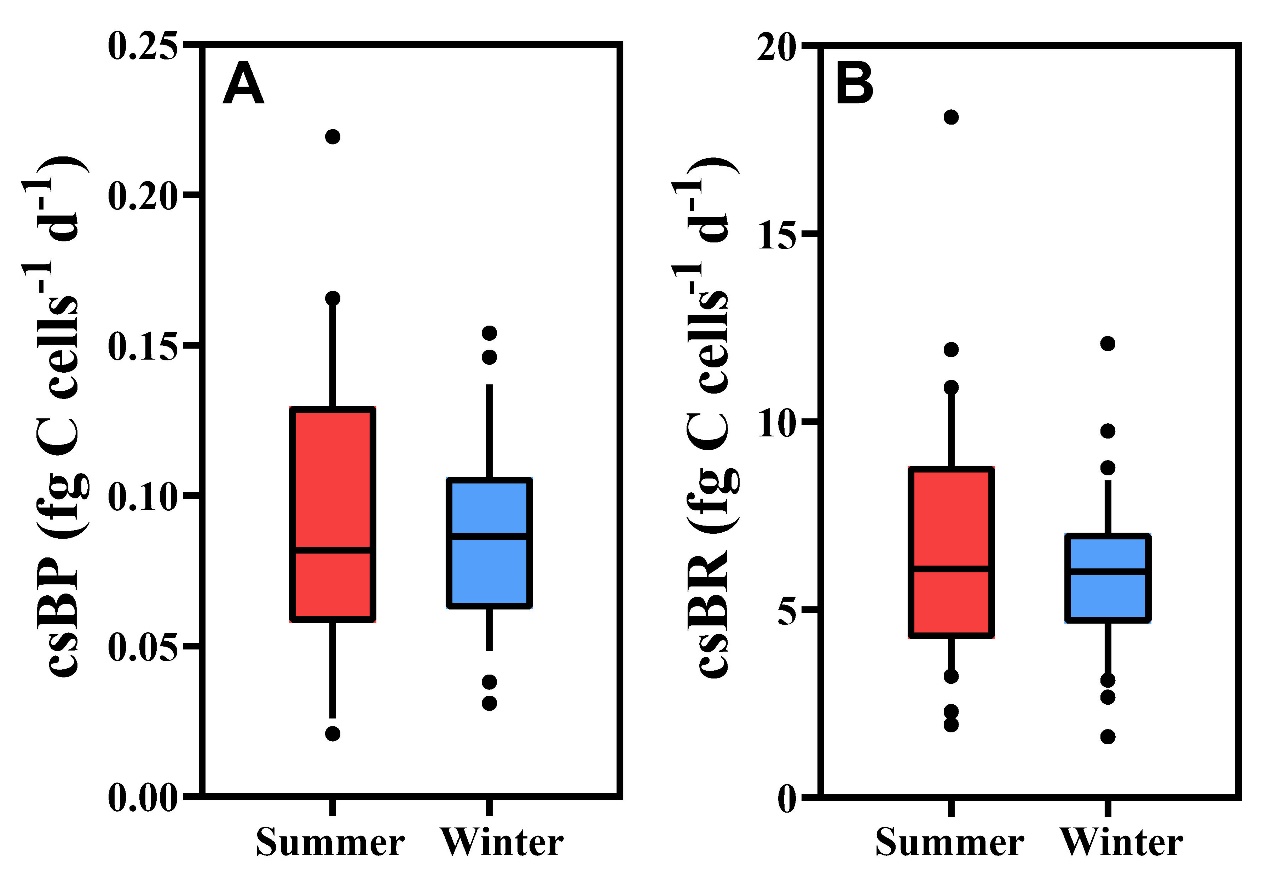


Fig. S2 The differences in bacterial metabolism between summer and winter include cell-specific bacterial production (csBP) (A) and cell-specific bacterial respiration (csBR) (B)


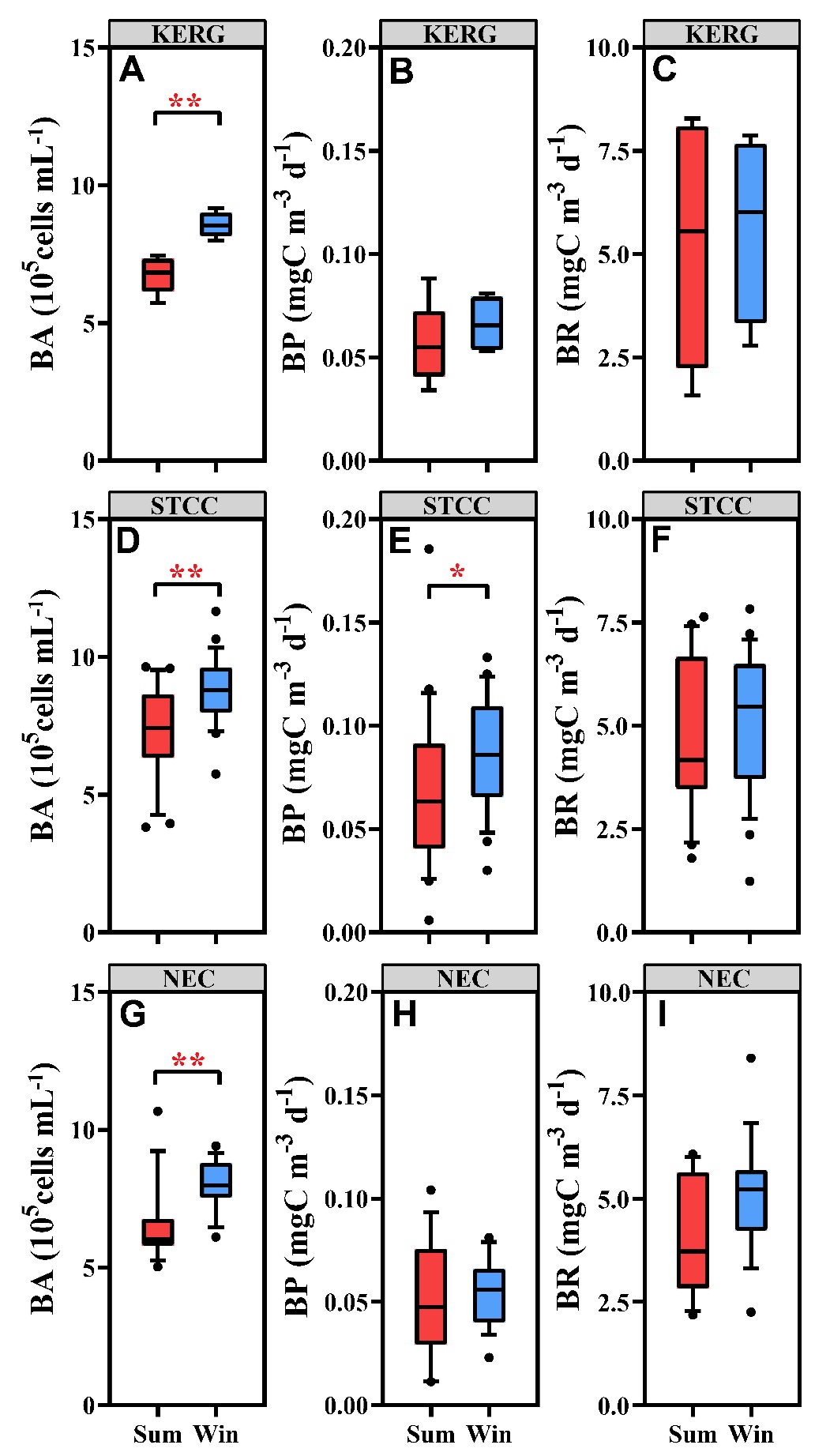


Fig. S3 The differences in bacterial metabolism between summer and winter among Kuroshio Extension region (KERG) (A-C), Subtropical Countercurrent (STCC) (D-F) and North Equatorial Current (NEC) (G-I) (Wilcoxon test, * *p* < 0.05, ** *p* < 0.001)


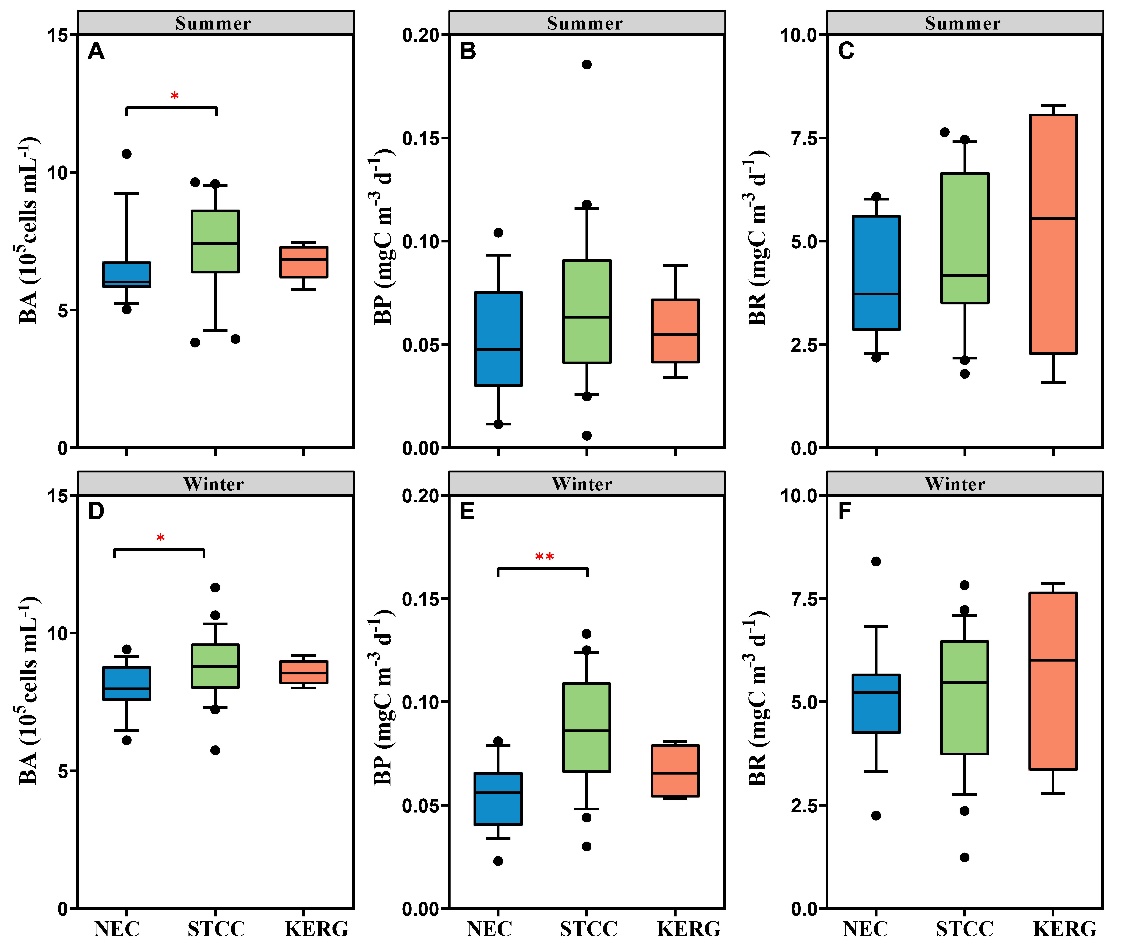


Fig. S4 The differences in bacterial metabolism among Kuroshio Extension region (KERG), Subtropical Countercurrent (STCC) and North Equatorial Current (NEC) between summer (A-C) and winter (D-F) (Wilcoxon test, * *p* < 0.05, ** *p* < 0.001)


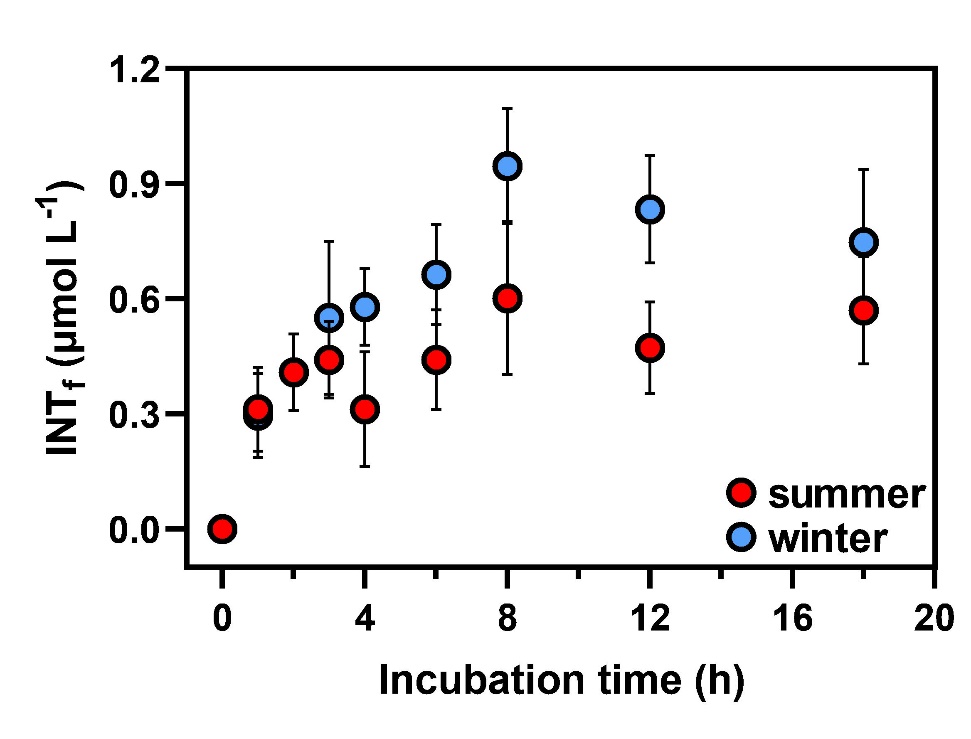


Fig. S5 The relationship between INT_f_ (formazan) reduction rates and incubation time at surface layers during summer and winter cruise in the NPSG


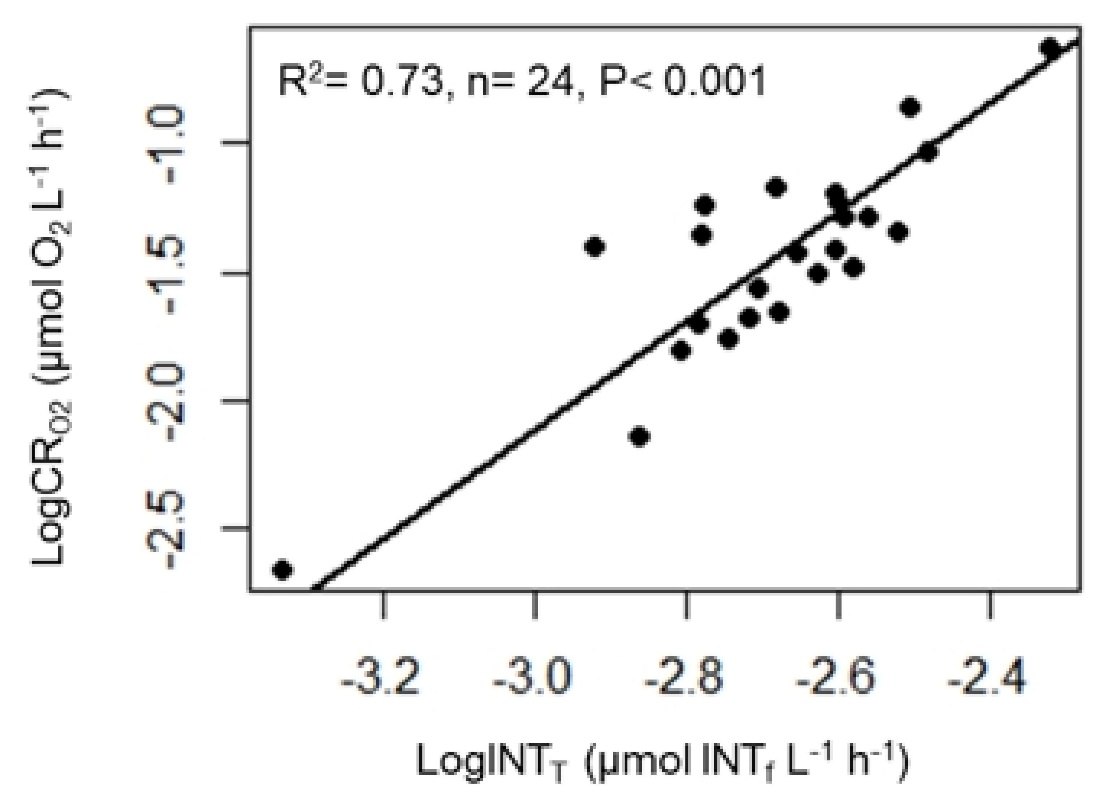


Fig. S6 Log-Log relationship between dissolved oxygen consumption (CR_O2_) measured by Winkler titration and INT reduction rate (INT_T_) measured by ETS method during summer and winter cruise in the NPSG
